# Genetic variation analysis of Guanling cattle based on whole-genome resequencing

**DOI:** 10.5713/ab.24.0181

**Published:** 2024-06-27

**Authors:** Longxin Xu, Xin Wang, Junda Wu, Hua Wang, Wenzhang Zhou, Jing Liu, Mengmeng Ni, Kaikai Zhang, Bo Yu, Ruiyi Lin

**Affiliations:** 1 Institute of Animal Husbandry and Veterinary Science, Guizhou Academy of Agricultural Sciences, Guiyang 550005, China; 2 College of Animal Sciences, Guizhou University, Guiyang 550000, China; 3 Guizhou Yellow Cattle Industry Group Co., Ltd, Guiyang 550001, China; 4 College of Animal Sciences, Fujian Agriculture and Forestry University, Fuzhou 350002, China

**Keywords:** Gene Screening, Guanling Cattle, Resequencing, Single Nucleotide Polymorphism (SNP)

## Abstract

**Objective:**

The objective of this study was to unravel the genetic traits of Guanling cattle, pinpoint genes advantageous for muscle growth, and lay a foundation for the preservation of genetic diversity and further analysis of regulation mechanism of important economic traits in local cattle breed.

**Methods:**

In this study, we sequenced the whole genome of 3 Guanling cattle in Guizhou province using the Illumina HiSeq cBo sequencing platform. And, high-multiplex polymerase chain reaction technology was employed to detect high-quality single nucleotide polymorphism (SNP) sites of other 55 Guanling cattle.

**Results:**

Our study identified 166,411 non-synonymous SNPs (nsSNPs) and 42,423 insertions and deletions (indels). Through SNP annotation, gene function enrichment analysis, and comparing with Simmental, Angus, and Limousin cattle, we identified six genes (*LEPR*, *AKAP9*, *SIX4*, *SPIDR*, *PRG4*, *FASN*) which are potentially influential on meat quality traits, playing crucial roles in muscle growth, fat metabolism, and bodily support. We also examined polymorphisms at seven SNP sites in Guanling cattle and found that all seven were in Hardy–Weinberg equilibrium.

**Conclusion:**

These findings suggested that these gene sites are stable and widespread in the Guanling cattle population. Our research lays the groundwork for future genetic enhancement and variety identification of Guanling cattle.

## INTRODUCTION

In the extensive history of cattle breeding in Guizhou, a diverse range of local cattle breeds has developed, with the Guanling cattle being a prominent example. These cattle primarily inhabit the vast mountainous regions within the Panjiang River basin, spanning across Guizhou, Yunnan, and northern Guangxi, with Guanling County being their most renowned habitat [1]. Guanling cattle represent a superior local breed in Guizhou Province and are among the 78 nationally protected local livestock breeds. They are highly regarded for their culinary, economic, and developmental potential. Previous research has employed biochemical and molecular techniques to analyze the genetic characteristics of the Guanling breed [2]. These studies have revealed significant genetic variation and growth potential. However, the advancement and utilization of these breeds have faced limitations attributed to the introduction of foreign breeds, crossbreeding enhancements, environmental degradation, and various natural factors, which have led to a decline in their population.

Advances in sequencing technologies have greatly facilitated comprehensive and in-depth genome analysis. And, sequencing of the bovine genome and HapMap projects have revealed a substantial number of genetic variations [3], with single nucleotide polymorphisms (SNPs) being the most extensively studied variant. SNPs serve as valuable tools for identifying genomic regions through association analysis, as they exhibit linkage disequilibrium with quantitative trait loci influencing target traits, a phenomenon observed in various animal species. For instance, Eck et al [4] identified 2.4 million SNPs in Holstein cattle using the Illumina HiSeq platform. Stothard et al [5] employed SOLiD technology to successfully map genomic variations between Black Angus bulls and Holstein bulls, identifying approximately 7 million SNPs and 790 copy number variations. The utilization of SNPs as selection criteria for meat traits in marker-assisted selection can significantly enhance cattle selection and breeding programs.

Recent studies on bovine genomic variation have been extensive, however, whole-genome studies on Guanling cattle have yet to be reported. Here, we resequenced the Guanling cattle and compared them with the Limousin, Simmental, and Angus breeds to reveal their genomic characteristics and variations. This study’s primary objective was to unravel the genetic traits of Guanling cattle, pinpoint genes advantageous for muscle growth, and provide essential genomic data to support further analysis of genetic mechanisms tied to economic traits and the preservation of cattle breed genetic diversity.

## MATERIALS AND METHODS

### Ethics approval

All animal experiments in the study were reviewed and approved by the Subcommittee of Experimetal Animal Ethics of Guizhou Academy of Agricultural Sciences.

### Animal samples

Blood samples of 58 Guanling cattle from the central production area in Guanling County were collected. To minimize the degree of relationship among individuals, adult bulls were randomly selected from farmers in different areas. And, three of them underwent whole-genome resequencing, while the remaining 55 cattle were analyzed via high-multiplex polymerase chain reaction (PCR) technology. Blood samples of one of each Simmental, Angus, and Limousin cattle (Guizhou Breeding Bull Station, Qianxi, Guizhou, China) were also collected for whole-genome resequencing as experimental controls.

### DNA library construction and sequencing

Genomic DNA was extracted from blood samples using the Blood Genomic DNA Extraction Kit (Tiangen Biotech Co., Ltd., Beijing, China) following the manufacturer’s instructions, and the extracted DNA was quality-inspected by Qubit2.0 and 0.8% agarose gel electrophoresis. Qualified DNA can be used for subsequent sequencing experiments. The genomic DNA was randomly interrupted by CovarisS2, recovered the DNA fragments (~300 bp) by electrophoresis, added the joint, according to the corresponding process shown in cBot User Guide, complete Cluster generation on the cBot equipped with the Illumina HiSeq sequencer according to the Illumina User Guide preparation sequencing reagent, and the flow cell with cluster was carried on the machine. The paired-end procedure was selected for two-end sequencing, which was controlled by data collection software provided by Illumina and used for real-time data analysis. The two-end sequencing length was 200 bp to give the final sequencing data. The DNA libraries were sequenced by Shanghai Biotechnology Corporation (Shanghai, China).

### Quality control and data filtering

In order to ensure the quality of the data, the sequencing raw data should go through quality control and data filtering. The quality of the raw data (three Guanling cattle, one of each Simmental, Angus, and Limousin cattle) is controlled by analyzing the composition and quality values of the bases (Table 1). The raw reads with some joint or low quality reads was filtered by fastp [6] to obtain high quality clean data, and subsequent analysis was based on clean data. Data filtering is mainly about removing paired reads with joints; removing paired reads with N base (N indicates uncertain base information) greater than 10%; removing paired reads with low quality (mass value Q 7) bases exceeding 30% of the total number of reads.

### Sequencing data alignment

Clean reads from each sample were aligned with the bovine reference genome (GCA_002263795.2) using Burrows–Wheeler Aligner 0.7.13 (BWA) [7] with the following parameters: “mem 4-k 32-M”. In this context, “-k” denotes the minimum seed length, and “-M” flags shorter split alignments as secondary. Sorting and deduplication were carried out using the SAMtools and Picard toolkits. SNP/indel detection was conducted using the Genomic Analysis Toolkit (GATK) HaplotypeCaller [8].

After obtaining SNP information from the samples, genotypes showing polymorphisms with the reference sequence were filtered using GATK’s variant filtration method with specified criteria (-Window 4, -filter “QD<2.0 ||FS>60.0|| MQ<40.0”, -G_filter “GQ<20”). To establish a high-confidence SNP/indel dataset, the identified SNPs and indels were called in variant call format and cross-referenced with the dbSNP database to identify novel variants. Finally, the snpEff tool was used for mutation annotation and statistical analysis.

### Functional gene enrichment and single nucleotide polymorphism selection

For the purpose of this study, Groups G26, G27, and G28, mean Guanling cattle, were collectively referred to as Group 1, while AG, LM, and XM (represent Limousin, Simmental, and Angus cattle, respectively) constituted Group 2. Mutations common to and distinct between these two groups were categorized separately. Subsequently, the gene loci associated with these mutations were subjected to gene ontology (GO) enrichment and pathway enrichment analysis via the DAVID database (https://david.ncif-crf.gov/).

Following enrichment analysis, SNP selection was initiated. First, group-specific SNP sites exhibiting consistent homozygous genotypes across three samples per group were chosen. Second, functional variant SNPs, including nonsynonymous mutations, premature terminations, intron-exon splice sites, and early starts, were selected. Third, variants with significant protein impacts were chosen, with exclusions made for those located on sex chromosomes. The proteins were encoded by those genes that are enriched. In the fourth step, group-specific SNPs obtained in the initial selection were filtered through a literature review to identify genes associated with meat quality traits [9–12]. Subsequently, functional SNPs were selected from those obtained in the fourth step, once again excluding variants on sex chromosomes. Finally, SNPs with high impact and associations with meat quality traits were evaluated for technical feasibility using Sanger sequencing based on PCR.

### Single nucleotide polymorphism detection

High-multiplex PCR technology was employed to detect high-quality SNP sites within a population of 55 Guanling cattle. Specific capture primers were designed, and the detection process was conducted by Shanghai Biowing Applied Biotechnology Co., Ltd. (Shanghai, China).

## RESULTS

### Sequencing and alignment

A quality assessment was performed on the initial data generated from whole-genome sequencing (WGS), which included the removal of potential PCR duplicates and realignment around insertions and deletions. After duplicate removal, an average data output of 26 Gb was obtained. The alignment rate of all the samples to the reference genome exceeded 99% (Table 2), with an average coverage of more than 97% (Table 3).

### Single nucleotide polymorphism detection

A total of 598,688 SNP sites were identified. After removing identical SNPs with different amino acid changes, 370,891 unique sites remained, 362,493 of which were located on autosomes, 8,169 on sex chromosomes, and 229 on mitochondria. The SNP density averaged one mutation per 374 bases, enabling the localization of various candidate genomic regions associated with economic traits. A comparison with the dbSNP database revealed 13,769 SNPs not present in the database, indicating that 2.3% of the identified SNPs were novel. Among them, 24,606 were homozygous SNPs (4.11%), and 574,082 were heterozygous SNPs (95.89%), resulting in a heterozygous-to-homozygous ratio of 23.33. Furthermore, the transition/transversion ratio (Ts/Tv) is a crucial metric for assessing random sequence errors in SNP quality. The empirical Ts/Tv value in WGS studies is >2.1 [13]. In this study, the Ts/Tv ratio was approximately 2.33, surpassing the empirical value and affirming the accuracy of the identified SNPs for further research.

### Functional annotation and potential functional exploration of non-synonymous single nucleotide polymorphisms

**Figure 1:**
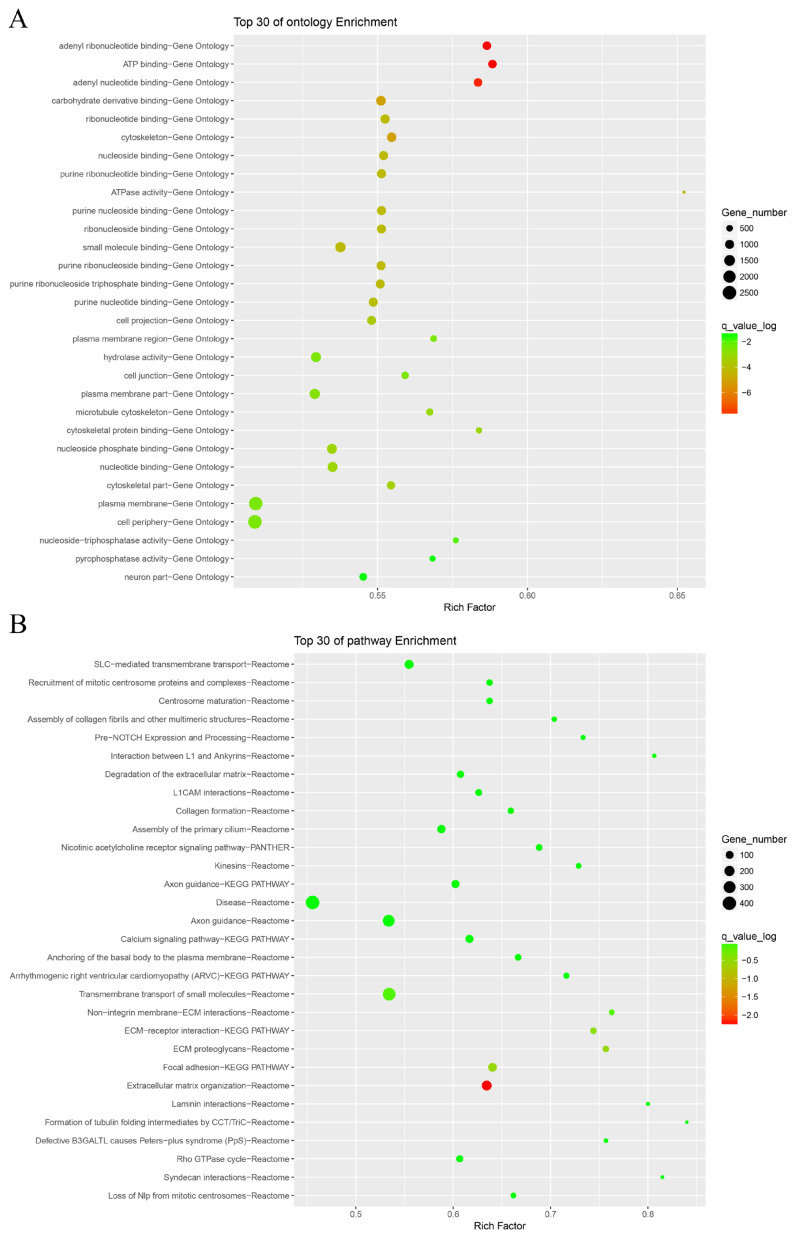
Enrichment analysis of nsSNPs. (A) Gene ontology enrichment of common SNPs between the two groups; (B) Kyoto encyclopedia of genes and genomes enrichment of common SNPs between the two groups. nsSNPs, non-synonymous single nucleotide polymorphisms; SNP, single nucleotide polymorphism.

A total of 166,411 non-synonymous SNPs (nsSNPs) were identified, and their mutations potentially play a pivotal role in altering economic traits in Guanling cattle. All of these mutations were annotated and subjected to GO functional enrichment and Kyoto encyclopedia of genes and genomes (KEGG) pathway enrichment analyses. GO functional enrichment analysis revealed 251 terms, mainly including adenyl ribonucleotide binding, ATP binding, adenyl nucleotide binding and so on (Figure 1A).

While KEGG pathway enrichment analysis revealed 60 pathways associated with genes whose expression was significantly enriched (p<0.05), including Extracellular matrix organization, Focal adhesion, extracellular matrix (ECM) proteoglycans, ECM-receptor interaction, Non-integrin membrane-ECM interactions, Transmembrane transport of small molecules and so on. Notably, the “Transmembrane transport of small molecules” pathway had the highest number of genes involved, with the highest enrichment found in the “Extracellular matrix organization” (Figure 1B).

### Indel detection

**Figure 2:**
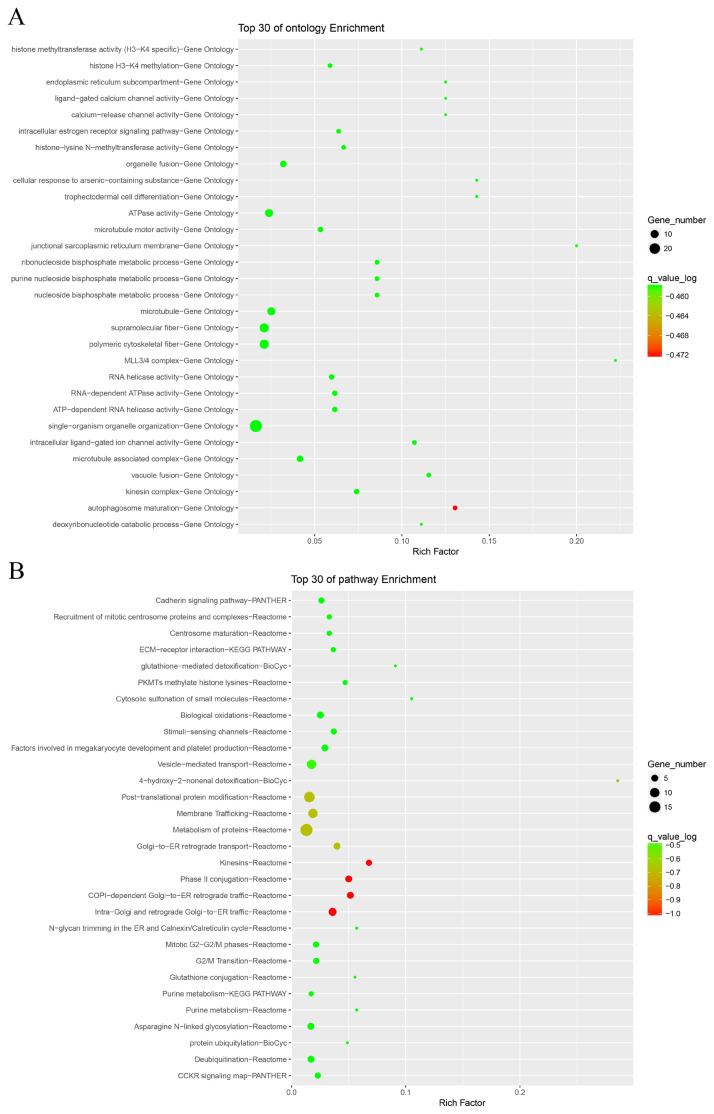
Enrichment analysis of indels. (A) Represents the gene ontology enrichment of common indels between the two groups; and (B) represents the Kyoto encyclopedia of genes and genomes enrichment of common indels between the two groups.

Typically, deletions and insertions over 50 base pairs (bp) are considered structural variations, while those under 50 bp are collectively referred to as insertions and deletions (indels) [14]. In Guanling cattle, a total of 42,423 indels were identified, comprising 22,101 deletions and 20,322 insertions, accounting for 52.09% and 47.91%, respectively. GO functional enrichment analysis yielded 105 terms, including autophagosome maturation, kinesin complex, vacuole fusion, microtubule associated complex, intracellular ligand-gated ion channel activity and so on (Figure 2A). After significant enrichment analysis of the KEGG pathways, 48 pathways were found to be notably enriched (p<0.05), including Intra-Golgi and retrograde Golgi-to-ER traffic, COPI-dependent Golgi-to-ER retrograde traffic, Kinesins and so on (Figure 2B).

### Candidate gene selection

Unique 1,520 homozygous SNP loci between the two groups were screened, excluding those on sex chromosomes and mitochondria. This led to the identification of 91 loci related to meat quality traits. We further selected loci with missense mutations and utilized the Ensemble database for annotating non-synonymous SNPs, resulting in the identification of 7 loci related to meat quality traits involving 6 different genes (Table 4).

### Single nucleotide polymorphism detection

To determine the distribution of the aforementioned 7 SNPs within the Guanling cattle population, high-multiplex PCR was employed for locus scanning in 55 cattle. The results (Table 5) showed that all 7 loci were in Hardy–Weinberg equilibrium. The identified mutation loci in the *LEPR*, *SIX4*, *SPIDR*, and *FASN* genes were dominant alleles in the Guanling cattle population.

## DISCUSSION

The number of Guanling cattle is small, so it is particularly important to select individuals which can represent varieties for sequencing. In order to avoid individual differences, more individuals are gathered at a low cost to reflect the population genetic diversity of Guanling cattle varieties. Therefore, three Guanling cattle samples were used for resequencing, and 55 Guanling cattle samples were used for further SNP detection. Finally, we get an average of 26 Gb raw data, 99.5% reads aligned to the reference genome, with high single base correctness, similar to the previous sequencing results of ordinary cattle [4,5]. High sequencing depth, and the detected variants are fully credible [15].

Here, we found 598,688 SNPs and 42,423 indels in 29 autosomes and X chromosomes. Of the total SNP, heterozygous SNPs 574,082 (95.89%), homozygous SNPs 24,606 (4.11%) and the ratio of heterozygous/homozygous SNPs was 23.33, significantly higher than that of Japanese cattle (1.24) [16] and Korean cattle (1.63) [17]. From a sequencing perspective, pure sequences and SNPs exhibit distinct characteristics. In mixed samples, the base types at a particular site are consistent with the reference genome. However, heterozygous SNPs indicate multiple base types at the same site across all mixed samples. Guanling cattle display a low homozygosity ratio for SNPs, suggesting high heterozygosity. This observation may reflect sequencing variations among individuals and could also be attributed to low levels of cattle breeding. Additionally, there appears to be increased gene communication between Guanling cattle and other breeds, potentially resulting in the loss of specific functional genes. Protecting the genetic diversity of Guanling cattle varieties is therefore of paramount importance. By comparing SNPs and indels, 13,769 new SNPs and 2,206 new indels were found, accounting for 2.3% of the total SNPs and 5.2% of the total indels, respectively. Due to the development of genome sequencing in recent years, more new SNPs and indels have been found, and the database is more and more perfect. So, the proportion of comparison is significantly increased, and the number of new discoveries gradually less. Most indels are short in length, with deletions ranging from 1 to 29 kb, insertions from 1 to 44 kb, the number of deletions and insertions concentrated in 1 to 10 bp, with 1 to 3 bp being the most, and similar phenomena are observed from human genome data [18]. In Guanling cattle data, nearly 85.6% insertions and 77.9% deletions were less than 5 kb. SNPs and indels detected on 29 autosomes are proportional to chromosome length and the expected results with the lowest X chromosome mutation rate of 4.61%, and on small population studies, the X chromosome has a lower mutation rate compared to the autosomes [19].

Guanling cattle are mainly used for cultivated land, and have gradually developed towards the direction of serving meat. The *LEPR* gene is located on bovine chromosome 3 and encodes a protein belonging to the cytokine receptor gp130 family. It plays a crucial role in regulating fat metabolism and is a novel hematopoietic pathway essential for normal lymphocyte production [20]. Previous research by Raza et al [21] investigated that three SNPs of *LEPR* gene were associated with backfat thickness, and intramuscular fat content in Chinese beef cattle breeds. Moreover, the expression level of *LEPR* was significant difference in the longissimus dorsi muscle of Yunling and Simmental cattle [22]. On bovine chromosome 4, the *AKAP9* gene, a member of the AKAP family, encodes a kinase anchoring protein. Raza et al [10] findings suggest that bovine *AKAP9* may be involved in regulating fat formation, growth traits, differentiation of adipose tissue, regeneration of skeletal muscle, and metabolism.

Like the bovine chromosome 10 gene, the *SIX4* gene, which is part of the Sine Oculis/Six gene family, plays a crucial role in skeletal muscle development. Genetic variations or deletions in *SIX4* can have implications for pituitary function [23]. Wang et al [24] research revealed significant correlations between three SNPs in the *SIX4* gene of Qinchuan cattle and body measurement traits, suggesting that *SIX4* is a candidate gene influencing cattle body size traits. Huang et al [25] demonstrated that *SPIDR* regulates the assembly or stability of RAD51/DMC1 on single-stranded DNA, a vital recombination factor in meiotic recombination in mammals. Zhang et al [26] demonstrated the significant effects of the *SPIDR* gene on growth parameters and carcass traits in 1,173 Chinese Simmental beef cattle.

Along bovine chromosome 16, the *PRG4* gene encodes a proteoglycan-like glycoprotein synthesized by various tissues, including joint cartilage, the meniscus, the synovial lining, and tendon cells [27]. Research by Abubacker et al [28] emphasized the importance of the intermolecular disulfide bond polymers of *PRG4* for its adsorption onto cartilage surfaces and function as a boundary lubricant. These findings suggested that *PRG4* may play a role in reducing friction in tissues and spaces of bovine knee joints, contributing to effective weight-bearing systems, particularly during weight gain in Guanling cattle.

Fatty acid synthase (FASN) encodes a versatile protein primarily responsible for catalyzing the synthesis of palmitic acid from acetyl-CoA and malonyl-CoA, facilitated by nicotinamide adenine dinucleotide phosphate (NADPH), resulting in the formation of long-chain saturated fatty acids [29]. Previous studies have identified *FASN* as a pivotal candidate gene influencing the composition of fat in both milk and meat [30,31]. Chu et al [32] observed significant variations in intramuscular fat content among Datong yaks based on different *FASN* gene genotypes. Individuals with the HH and HG genotypes exhibited notably greater intramuscular fat content than did those with the GG genotype [32]. *FASN* mRNA expression levels in subcutaneous fat and abdominal fat in Yan yellow cattle were significantly higher than that in Yanbian yellow cattle [33]. Genome-wide association analysis revealed that g.841G>C SNP of *FASN* gene showed significant associations with the percentages of C14:0, C14:1, C16:1, and C18:1 at 5% genome-wide significance level in Japanese Black cattle [34]. In our study, we screened seven SNP sites spanning six genes within the Guanling cattle population. The dominant alleles in this population were found to be A at rs43347904 within the *LEPR* gene, A at rs133120166, T at rs109170670 within the *SIX4* gene, C at rs208094969 within the *SPIDR* gene, and A at rs715140536 within the *FASN* gene. However, the biological functions of these mutation sites require further investigation.

## CONCLUSION

This study conducted genome-wide genotyping of Guanling cattle, which yielded an abundance of genetic markers. These markers, which are crucial for selection purposes, serve as powerful tools for enhancing the breeding and management of Guanling cattle, offering valuable insights for breeding decisions. The integration of resequencing with advanced breeding techniques to identify gene polymorphisms and characteristics holds great promise in elevating the breeding standards of economic animals, with a specific focus on improving growth and meat quality traits. Our research successfully pinpointed several genes which may influence meat quality traits, thus contributing essential knowledge about the variety identification and optimal breeding strategies for the Guanling cattle breed.

## Figures and Tables

**Table 1:** Quality evaluation of sequencing data

Sample name	Total reads	Total bases	Q≥20 (%)	Q≥30 (%)
LM	210,571,554	31,585,733,100	97.26	92.63
XM	178,721,090	26,808,163,500	97.14	92.38
AG	159,908,598	23,986,289,700	97.37	92.80
G26	149,728,482	22,459,272,300	97.27	92.67
G27	178,927,018	26,839,052,700	97.36	92.83
G28	165,752,804	24,862,920,600	97.35	92.78

LM, XM, AG represent Limousin, Simmental, and Angus cattle, respectively. G26, G27, G28 mean Guanling cattle.

**Table 2:** The result of sample mapping

Sample	Total reads	Mapped reads	Mapped ratio (%)	Duplicates	Duplicates ratio (%)
LM	209,620,678	208,574,512	99.50	22,771,674	10.86
XM	177,891,074	176,957,213	99.48	18,280,590	10.28
AG	159,306,970	158,784,047	99.67	16,165,746	10.15
G26	149,109,024	148,260,266	99.43	16,347,741	10.96
G27	178,202,188	177,363,966	99.53	20,863,565	11.71
G28	165,038,540	164,277,230	99.54	18,533,568	11.23

LM, XM, AG represent Limousin, Simmental, and Angus cattle, respectively. G26, G27, G28 mean Guanling cattle.

**Table 3:** The result of sequencing depth and coverage

Sample	Reference length (bp)	Covered base	Coverage (%)	Depth
LM	2,715,853,792	2,668,752,045	98.27	10.02
XM	2,715,853,792	2,660,757,927	97.97	8.53
AG	2,715,853,792	2,651,574,588	97.63	7.69
G26	2,715,853,792	2,641,397,151	97.26	7.09
G27	2,715,853,792	2,650,192,327	97.58	8.39
G28	2,715,853,792	2,649,665,821	97.56	7.81

LM, XM, AG represent Limousin, Simmental, and Angus cattle, respectively. G26, G27, G28 mean Guanling cattle.

**Table 4:** Single nucleotide polymorphism associated with meat quality traits

Chr	Pos	Ref	Alt	Func_site	Functional_Class	Codon_Change	Gene_Name
3	79,817,216	G	A	NON_SYNONYMOUS_CODING	MISSENSE	tCc/tTc	*LEPR*
4	9,411,235	C	A	NON_SYNONYMOUS_CODING	MISSENSE	gaC/gaA	*AKAP9*
10	72,877,478	G	A	NON_SYNONYMOUS_CODING	MISSENSE	Cca/Tca	*SIX4*
10	72,877,640	C	T	NON_SYNONYMOUS_CODING	MISSENSE	Gct/Act	*SIX4*
14	19,224,954	G	C	NON_SYNONYMOUS_CODING	MISSENSE	Cac/Gac	*SPIDR*
16	67,393,711	C	T	NON_SYNONYMOUS_CODING	MISSENSE	Cca/Tca	*PRG4*
19	50,782,773	G	A	NON_SYNONYMOUS_CODING	MISSENSE	Gcc/Acc	*FASN*

Chr, stands for chromosome ID; Pos, for the position of the variant on the chromosome; Ref, for the reference base; Alt, for the base differing from the reference; Func_site, for the annotation of the mutation site in the gene functional region; Functional_Class, for the type of mutation; Codon_Change, for the codon transformation; Gene_Name, for the name of the gene where the locus is located.

**Table 5:** Detection of single nucleotide polymorphism locus

Gene	SNP	Genotype	Genotypic frequency	Allele	Allele frequency	Hardy-weiberg equilibrium	

						X^2^	P
*LEPR*	rs43347904	GG	0	G	0.05	0.18	0.91
		GA	0.11	A	0.95		
		AA	0.89				
*AKAP9*	rs434631905	CC	0.4	C	0.57	4.76	0.09
		CA	0.35	A	0.43		
		AA	0.25				
*SIX4*	rs133120166	GG	0.07	G	0.18	3.91	0.14
		GA	0.22	A	0.82		
		AA	0.71				
*SIX4*	rs109170670	CC	0	C	0.16	2.11	0.35
		CT	0.33	T	0.84		
		TT	0.67				
*SPIDR*	rs208094969	GG	0.07	G	0.22	0.19	0.55
		GC	0.29	C	0.78		
		CC	0.64				
*PRG4*	rs109282393	CC	0.44	C	0.66	0.02	0.99
		CT	0.45	T	0.34		
		TT	0.11				
*FASN*	rs715140536	GG	0.03	G	0.15	0.5	0.78
		GA	0.24	A	0.85		
		AA	0.73				

SNP, single nucleotide polymorphism.
